# Chronic Myeloid Leukemia: Atypical Presentation and Diagnostic Pitfall in the Workup of Isolated Thrombocytosis

**DOI:** 10.7759/cureus.8498

**Published:** 2020-06-07

**Authors:** Dawood Findakly, Waqas Arslan

**Affiliations:** 1 Internal Medicine, Creighton University Arizona Health Education Alliance/Valleywise Health Medical Center, Phoenix, USA; 2 Hematology and Oncology, Creighton University Arizona Health Education Alliance/Valleywise Health, Phoenix, USA; 3 Hematology and Oncology, Creighton University Maricopa Medical Center, Phoenix, USA

**Keywords:** isolated thrombocytosis, chronic myeloid leukemia, tyrosine kinase inhibitor, bcr-abl positive, philadelphia chromosome

## Abstract

Chronic myeloid leukemia (CML) is one of the classic types of myeloproliferative neoplasms. It typically manifests with leukocytosis, but rarely with isolated thrombocytosis. Here we describe a unique case of isolated thrombocytosis as an initial presentation of CML in a 21-year-old woman, where the BCR-ABL1 fusion gene was detected in bone marrow (BM) aspiration and biopsy specimen after a negative peripheral blood (PB) fluorescence in situ hybridization testing. It is crucial to pursue workup for patients with isolated thrombocytosis through testing for the presence of the BCR-ABL fusion gene or the Philadephia chromosome in both PB and the BM in order to distinguish CML from essential thrombocythemia.

## Introduction

The incidence of chronic myeloid leukemia (CML) in the United States is less than 5,000 cases per year [1]. CML usually presents with marked leukocytosis, but very rarely with an isolated, marked thrombocytosis [2]. Here we present a case of a 21-year-old woman who was discovered to have isolated thrombocytosis incidentally upon evaluation for vasovagal syncope. Her initial peripheral blood (PB) fluorescence in situ hybridization (FISH) testing for the BCR‐ABL gene was negative, but subsequent bone marrow (BM) aspiration and biopsy were consistent with CML. This case describes the perplexity in reaching an accurate diagnosis in this subset of patients where diagnosis might be missed and underscores how CML constitutes an essential differential diagnosis in patients presenting with isolated thrombocytosis.

## Case presentation

**Figure 1:**
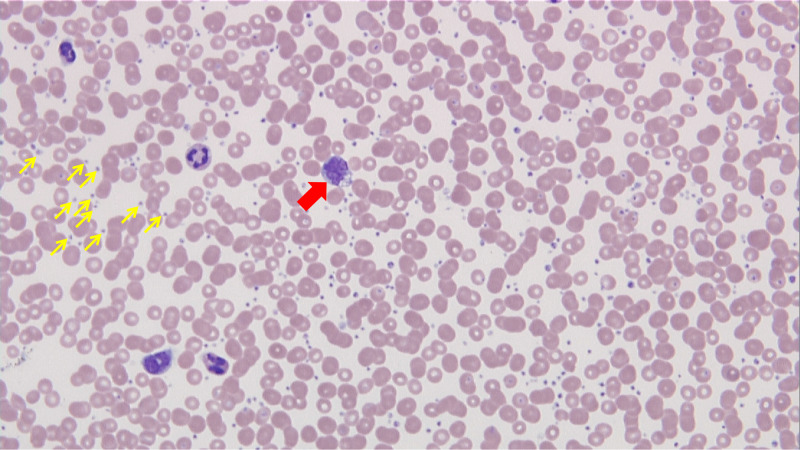
The PB smear exhibits markedly increased PLT (yellow arrows), anisopoikilocytosis without evidence of eosinophilia or basophilia. A rare immature monocyte displayed (red arrow) could rarely be seen. PB: peripheral blood; PLT: platelet

A 21-year-old previously healthy woman was referred to the hematology clinic for further workup of thrombocytosis, which was found upon evaluation for vasovagal syncope. Upon her presentation, vital signs were stable and physical examination was unremarkable. Laboratory workup was relevant for a white blood cell (WBC) count of 7.2×10^3/µL, with 56% neutrophils, 33% lymphocytes, 7% monocytes, 1% basophils, and 0.2% eosinophils, hemoglobin of 13.3×10^3/µL, platelets (PLT) of 764 K/µL, and minimally elevated erythrocyte sedimentation rate at 39 mm/hr. Peripheral smear confirmed thrombocytosis, and the patient was started on daily low-dose aspirin (Figure 1).

**Figure 2:**
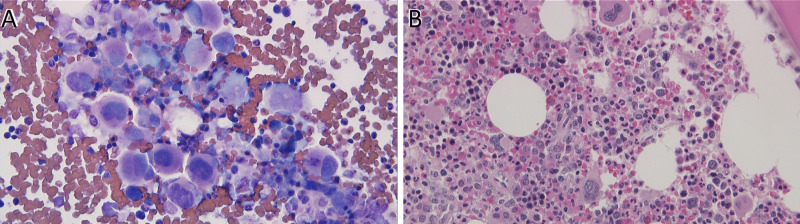
(A) BM aspirate smear; (B) BM core biopsy demonstrating an overall cellularity of approximately 65%-70%. The megakaryocytes are markedly increased with clustered numerous small and unilobed megakaryocytes appreciated. Moreover, the myeloid to erythroid ratio is at approximately 3:1 with a relative decrease in erythroid precursors. BM: bone marrow

Meanwhile, the thrombocytosis workup for myeloproliferative disorders (MPDs) with PB JAK2 on exon 12 and 14, calreticulin (CALR), and thrombopoietin receptor (MPL) genes were not mutated. Moreover, PB FISH for BCR‐ABL was negative, and a CT scan of the abdomen and pelvis was performed and was unremarkable. As PLT count continued to rise, reaching 1,096 K/µL, the hematology consultant recommended bone marrow aspirate and biopsy; which revealed overall cellularity of approximately 65%-70% with 14% segmented neutrophils, 10% bands, 7% metamyelocytes, 10% myelocytes, 0% progranulocytes, 3% blasts, 3% monocytes, 4% eosinophils, 0% basophils, 16% lymphocytes, 2% plasma cells, and 28% nucleated red blood cells (Figure 2). Moreover, bone marrow molecular biology analysis of the BCR/ABL fusion gene revealed a translocation t(9;22) BCR-ABL1 consistent with CML. 

**Figure 3:**
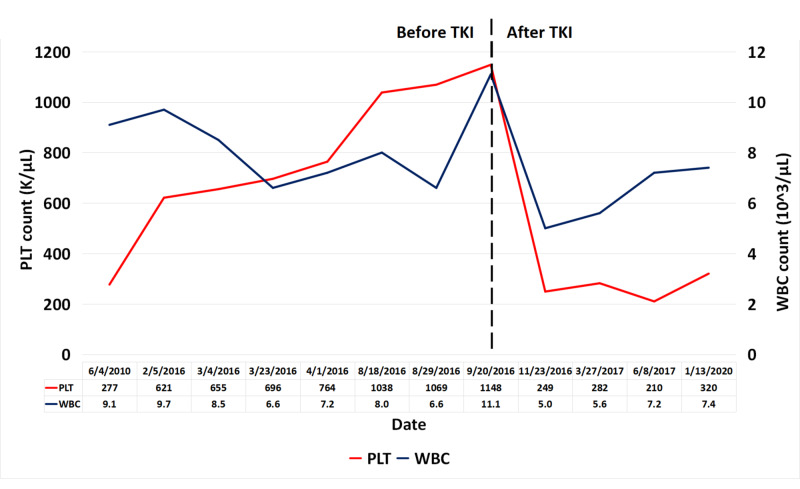
Timeline for PLT and WBC trend during follow-up, before and after TKI (dasatinib) therapy. The x-axis constitutes the date when testing was performed, the left y-axis constitutes PLT count, and the right y-axis constitutes WBC count. The vertical black hatched line indicates the starting point of TKI (dasatinib) treatment. PLT: platelet; WBC: white blood cell; TKI: tyrosine kinase inhibitor

Subsequently, the patient was initiated on a second-generation tyrosine kinase inhibitor (TKI), dasatinib, achieving a complete cytogenetic and molecular remission (Figure 3). The patient continues in remission at 32 months follow-up and is being evaluated for stem cell transplant.

## Discussion

CML incidence in the United States is approximately 1.3 cases per 100,000 per year [1]. Patients with CML are, in many cases, asymptomatic at diagnosis and only incidentally detected upon abnormalities found on routine blood testing. CML is the most common condition in the spectrum of MPDs [3]. It typically presents with leukocytosis, with elevated immature granulocytes, basophilia, and eosinophilia in the PB [3,4].

This atypical CML presentation with an isolated thrombocytosis is a rare entity [5]. Isolated thrombocytosis could lead to a misdiagnosis of essential thrombocythemia (ET) [4]. Thrombocytosis, described as a platelet count of more than 450×10^9^/L, is common in chronic MPDs [3]. Thrombocytosis could be reactive or it may be paraneoplastic where it could be triggered by altered immune response to MPDs, including polycythemia vera, CML, or ET [4,6]. Severe thrombocytosis, with a platelet count of more than 1,000×10^9^/L, is exceptionally rare to be found upon initial laboratory workup in newly diagnosed patients with CML [3]. Every patient presenting with isolated thrombocytosis should be tested to rule out CML [7]. CML is characterized by persistently enhanced Philadelphia positive (Ph+) chromosome, which is an active tyrosine kinase [8,9]. BCR-ABL has tyrosine kinase activity, which promotes cell proliferation and enhances resistance to apoptosis [9].

Despite the increasing prevalence of CML cases, TKI chemotherapy remains successful in improving survival outcomes [10]. BM specimens are superior to PB specimens in detecting BCR-ABL fusions [11]. Atypical CML presentation, with isolated thrombocytosis, should be considered in patients presenting with isolated marked thrombocytosis, as it signifies a very poor prognosis with a median overall survival of 24 months [12].

## Conclusions

Given its reduced survival outcomes, atypical presentation of CML should remain high in the differential diagnosis when evaluating patients with significant thrombocytosis, even when WBC count is normal. These patients should not be misdiagnosed as ET, and providers should pursue genetic testing for the BCR/ABL gene in both PB and BM in order to circumvent delayed or missed diagnosis, which could ultimately interfere with optimal medical treatment and prognosis. Further research is necessary to evaluate the clinicopathologic characteristics, treatments, and outcomes of CML patients presenting with isolated marked thrombocytosis.
